# Trends in myopia prevalence and projected visual impairment in Western Europe: a pooled analysis of Dutch population-based cohorts (1900–2000)

**DOI:** 10.1136/bmjph-2024-002307

**Published:** 2025-09-29

**Authors:** Sander Kneepkens, Clair A Enthoven, Jan Roelof Polling, Victor A de Vries, J Willem Lodewijk Tideman, Caroline Klaver, Eye Epidemiology research group, P T V M de Jong

**Affiliations:** 1 Department of Ophthalmology, Erasmus MC University Medical Center Rotterdam, Rotterdam, The Netherlands; 2 The Generation R Study Group, Erasmus MC University Medical Center Rotterdam, Rotterdam, The Netherlands; 3 Department of Child and Adolescent Psychiatry, Erasmus MC University Medical Center Rotterdam, Rotterdam, The Netherlands; 4 Department of Epidemiology, Erasmus MC University Medical Center Rotterdam, Rotterdam, The Netherlands; 5 Ophthalmology, Martini Hospital, Groningen, The Netherlands; 6 Department of Ophthalmology, Radboud University Medical Center, Nijmegen, The Netherlands; 7 Institute of Molecular and Clinical Ophthalmology Basel, Basel, Switzerland

**Keywords:** Epidemiology, Public Health, Preventive Medicine, Causality, Risk Assessment

## Abstract

**Importance:**

The global prevalence of myopia, a major cause of visual impairment and blindness, is on the rise. Understanding its trajectory and contributing factors in Europe is essential for implementing effective public health interventions.

**Objective:**

To assess the trend of myopia prevalence in Europe over the last century, examine the role of education across generations, and project future rates of myopia-related visual impairment and blindness.

**Design:**

This observational study used data from population-based cohorts in Rotterdam, Netherlands, including the Rotterdam Study I–IV, Generation R and Generation R Next.

**Setting:**

Population-based cohort studies conducted in Rotterdam, Netherlands.

**Participants:**

A total of 18 686 individuals born between 1900 and 2000, who underwent comprehensive ophthalmologic examinations.

**Exposures:**

Participants were stratified by birth year (1900–1920, 1920–1940, 1940–1960, 1960–1980 and 1980–2000) and analysed for associations between birth year and myopia prevalence, axial length (AL) and spherical equivalent (SE). The potential mediating and moderating role of education was examined using a four-way decomposition approach. Prevalence of severe visual impairment caused by myopia-related complications was estimated for the Netherlands and Europe.

**Main outcomes and measures:**

The primary outcomes were the prevalence of myopia (SE ≤−0.5 diopters (D)) and high myopia (SE ≤−6 D), and the projected prevalence of severe visual impairment due to myopia up to 2075.

**Results:**

Myopia prevalence increased 2.5 times (from 22% to 56%) and high myopia 3.5 times (from 2% to 7%) between 1900 and 2000. Compared with individuals born in 1900–1920, those born in 1980–2000 had significantly higher odds of being myopic (OR 4.79 (95% CI 3.72 to 6.18)) and highly myopic (OR 3.30 (95% CI 1.79 to 6.21)). Mean AL increased by 0.9 mm in men and 0.8 mm in women over the century. Education significantly mediated the association between birth year and myopia. By 2075, the prevalence of severe visual impairment or blindness due to myopia in Europe is projected to rise from 0.12% in 2015 to 0.84%, causing an estimated 3.6 million severely visually impaired.

**Conclusions and relevance:**

Myopia prevalence has risen dramatically in Europe, now affecting over 50% of young adults, with a corresponding increase in eye elongation over time. The burden of myopia-related visual impairment is expected to rise sharply as the population ages. By 2075, 0.84% of the European population is projected to be severely visually impaired or blind due to myopia-related complications. These findings underscore the urgent need for targeted public health interventions.

**WHAT IS ALREADY KNOWN ON THIS TOPIC**

- Myopia prevalence is rising globally, and in Europe, evidence for generational trends has been inconsistent, often based on heterogeneous, cross-sectional data without standardised measures such as axial length.

**WHAT THIS STUDY ADDS**

- This study demonstrates a 2.5-fold increase in myopia and a 3.5-fold increase in high myopia in the Netherlands from 1900 to 2000, using pooled, individual-level data from six population-based cohorts. This will lead to a sixfold increase in visual impairment due to myopia by 2075.

**HOW THIS STUDY MIGHT AFFECT RESEARCH, PRACTICE OR POLICY**

- These findings highlight the growing public health burden of myopia in Western Europe. The projected rise in vision impairment due to myopia underlines the urgency for implementing prevention and early detection strategies in clinical and policy frameworks.

## Introduction

 Myopia, the most common eye condition worldwide, is a refractive error which can be easily corrected by optical measures. Although the absence of correction is the most common cause of visual impairment globally, the accessibility to glasses, contact lenses and refractive surgery in high-income countries did not resolve myopia’s risks.^1^ The morbidity of myopia arises from elongation of the eye, which can lead to complications such as retinal detachment, glaucoma and myopic macular degeneration, conditions which form serious threats to central vision.^2^ While high myopes with particularly elongated eyes face the greatest risk of complications, those with mild and moderate myopia impose the highest burden to clinical care due to their higher prevalence in the population.^2 3^

Although the growing myopia epidemic in the highly educated world has been well documented, current speculations on trends are based on heterogeneous data from different regions collected at different time points.^4^,^6^ In contrast to the consistent reports from East Asia on the >80% frequency of myopia in young adults, the reported prevalence in older adults from this region varies widely.^7^ In Europe, consistent longitudinal studies on myopia are also scarce; reported frequencies vary from 47% in 20-year-olds from the UK, 35% in 35+ year-olds from Germany, to 14% in 60+ year-olds from Greece.^6 8 9^ Most reports were based on summary data of spherical equivalent (SE), which had been measured using different technologies.^4 6 10^ Trend speculations did not focus on axial length (AL), despite it being recognised as the most crucial determinant of myopia’s morbidity.^2 11^

Therefore, in this study, we aim to determine the change in myopia prevalence and AL across the last century in the Netherlands, using individual-level data from six large population-based cohort studies. These cohorts were selected because they included adult participants from a similar region with similar recruitment methods within the same institute. As AL and SE are not subject to significant changes in adulthood, this allowed for comparisons across generations.^12 13^ Our primary research questions were: has myopia prevalence changed over time; has AL changed consistently with refractive error and to what extent can these trends be explained by changes in educational attainment? Finally, we project the future burden of myopia-related visual impairment using established risk estimates, age-specific population projections and birth year-specific prevalence. These projections are important for policymakers, as they carry substantial medical and financial implications and may further highlight the urgency of implementing myopia control measures in children.

## Material and methods

### Study population

Participants from six Dutch studies were included in this analysis: older adults from the Rotterdam Study I–IV, and mothers from the birth cohort studies Generation R and Generation R Next. Detailed descriptions of the studies have been provided elsewhere.^14^,^16^ In brief, the Rotterdam Study is a population-based prospective study consisting of 4 cohorts of older adults living in a suburb of Rotterdam, the Netherlands. Cohort enrolment: the Generation R and Generation R Next studies are both population-based prospective cohort studies of pregnant women and their offspring residing within the Rotterdam city limits. Participants were enrolled between 1990 and 2021 across six Dutch cohort studies: Rotterdam Study I (1990–1993), Rotterdam Study II (2000–2001), Rotterdam Study III (2006–2008), Rotterdam Study IV (2012–2014), Generation R (2002–2006) and Generation R Next (2017–2021).^14^,^16^ No participant overlap occurred between these studies. Participants were included if they were aged ≥24 years, based on the assumption that refractive error stabilises in adulthood, and had either refractive error or biometry data available at a single time point. For individuals with longitudinal data, only the earliest available measurement was used.^12 13^ In total, we included 18 856 participants: 12 804 adults born between 1900 and 1976 from Rotterdam Study I–IV, 5632 adult women (ie, mothers) born between 1957 and 1989 from Generation R and 420 adult women born between 1975 and 2000 from Generation R Next. A detailed flow chart of participant selection and cohort contribution can be found in online supplemental figure 1. In addition, the demographics of each cohort are described in online supplemental table 1.

### Eye data collection

Automated refractive error measurements were collected (n=15 009). SE was calculated as the spherical refraction +1/2 of the cylinder for both eyes and subsequently averaged over both eyes. Myopia was defined as SE ≤−0.5 dioptres (D). Participants with cataract or pseudophakia were excluded from the refractive error analyses to avoid confounding from lens-induced refractive changes. This left 84% of the initial sample available for analysis (n=12 676 out of 15 009). Ocular biometry was measured in all studies (n=14 257). For AL, 3–5 measurements per eye were averaged to obtain the mean AL.

### Covariate data collection

Demographic, socioeconomic and lifestyle information was collected through extensive questionnaires and interviews. Level of highest attained education was grouped in accordance with the UNESCO international standard classification of education (ISCED 2011) as low (ISCED level 0–2), medium (ISCED level 3–5) and high (ISCED level 6–8).^17^ Anthropometry measures were collected during baseline visits at the research centre.

### Statistical methods

The study population was stratified into categories of birth year: 1900–1920, 1920–1940, 1940–1960, 1960–1980 and 1980–2000, see online supplemental table 2. Between-group comparisons were conducted using Student’s t-test for continuous measures, χ^2^ tests for dichotomous measures and analysis of variance with Tukey post hoc analysis for categorical measures with more than two groups. To analyse the association between categories of birth year and myopia prevalence, we used a logistic regression model corrected for sex. Linear regression models were applied to examine the relationship between SE and birth year, corrected for sex, as well as AL and birth year, corrected for sex and body height. Interaction terms between birth year and sex and sex and body height were used to assess potential effect modification.

We assessed the potentially mediating and moderating effects of education on the association between birth year and myopia prevalence, and between birth year and AL, using the four-way decomposition approach developed by VanderWeele^18^ (online supplemental figure 2). This method decomposes the total effect of birth year on myopia prevalence into the following components: (1) controlled direct effect (CDE), that is, birth year determines myopia prevalence independent of education; (2) reference interaction effect (INTref), that is, additive interaction effect of birth year and education evaluated at the reference (=low) level of education; (3) mediated interaction, that is, additive interaction effect between birth year and education evaluated at varying levels of education and (4) pure indirect effect (PIE), that is, the effect of birth year on myopia prevalence operates solely through education. As the model allows the mediator to be only dichotomous or continuous,^19^ we analysed education as low versus high, intermediate versus high and low versus intermediate. CIs for mediation effects were calculated using 1000 bootstrap resampling. All analyses were performed using complete case analysis; participants with missing covariate data were excluded. To estimate the impact of increasing myopia prevalence, we estimated the prevalence of visual impairment due to myopia in the Netherlands and in Europe for 2015, 2035, 2055 and 2075. First, we estimated the number of myopic individuals aged 75 years at this time using the prevalence rates reported in the current study and the population projections from both the Dutch Central Bureau of Statistics and Eurostat, the statistical office of the European Union.^20 21^ The cumulative risk of visual impairment and blindness, as defined according to WHO criteria, has been reported to be 39% for high myopes (SE <-6D) and 3.8% for intermediate myopes (between −0.5 and −6 D) at age 75.^11^ These cumulative risks, combined with the estimated number of (high) myopes aged 75+ years, were used to calculate the projected number of persons who will become visually impaired due to (high) myopia (online supplemental table 5).

## Results

### Demographics

Demographics and general characteristics are provided in table 1, categorised by 20-year birth year groups. The details of demographics per study cohort are provided in online supplemental table 1. Each group had a higher proportion of females, especially in the 1960–1980 (88% female) and 1980–2000 (100% female) groups, which included mothers from Generation R and Generation R Next. Mean age at examination varied significantly, with older participants in earlier groups. Education level also varied significantly (p<0.001), with low education decreasing from 64% (1900–1920) to 8% (1980–2000), and medium and high education increasing from 27% to 51% and from 9% to 41%, respectively. Height and AL increased significantly over time. Men’s height increased from 173.1 cm (1900) to 181.4 cm (1980), p<0.001, and AL from 23.4 mm to 24.2 mm, p<0.001. Women’s height increased from 159.3 cm (1900) to 166.5 cm (2000), p<0.001, and AL from 23.0 mm to 23.6 mm, p<0.001. Mean SE decreased from +1.0 D to −1.5 D; p<0.001 (table 1).

**Table 1:** Demographics divided into 20-year birth year groups, presented as mean±SD or total number (proportion)

	Demographics					
	1900–1920	1920–1940	1940–1960	1960–1980	1980–2000	P value*
Participants	908	5067	4862	6680	814	
Age (years)†	76.2±4.8	65.0±6.4	57.4±5.8	41.7±6.9	30.6±4.1	<0.001
Sex (female)	558 (62%)	2829 (56%)	2721 (56%)	5898 (88%)	814 (100%)	
Education level (%)						<0.001
Low	585 (64%)	2951 (58%)	2353 (48%)	1264 (19%)	60 (7%)	
Medium	244 (27%)	1482 (29%)	1228 (25%)	2073 (31%)	391 (48%)	
High	79 (9%)	634 (13%)	1281 (26%)	3343 (50%)	363 (45%)	
Height (cm)						<0.001
Male	173.1 (±6.1)	175.6 (±6.6)	178.0 (±6.9)	181.4 (±7.4)	NA	
Female	159.3 (±6.3)	162.7 (±6.1)	164.3 (±6.4)	167.8 (±7.3)	166.5 (±7.2)	
Axial length (mm)						<0.001
Male	23.4 (±1.0)	23.8 (±1.2)	24.1 (±1.3)	24.2 (±1.2)	NA	
Female	23.0 (±1.0)	23.3 (±1.2)	23.5 (±1.2)	23.7 (±1.2)	23.6 (±1.2)	
SE (D)						
Male	0.7 (±2.5)	0.8 (±2.4)	−0.3 (±2.5)	−0.6 (±2.3)		<0.001
Female	1.2 (±2.6)	0.9 (±2.5)	−0.1 (±2.6)	−0.6 (±2.5)	−1.5 (±2.4)	<0.001

* Analysis of variance corrected for gender differences was used to test significance for continuous variables and χ2 squared test for categorical variables.

† Mean age at time of examination.

### Temporal trend of myopia prevalence

**Figure 1:**
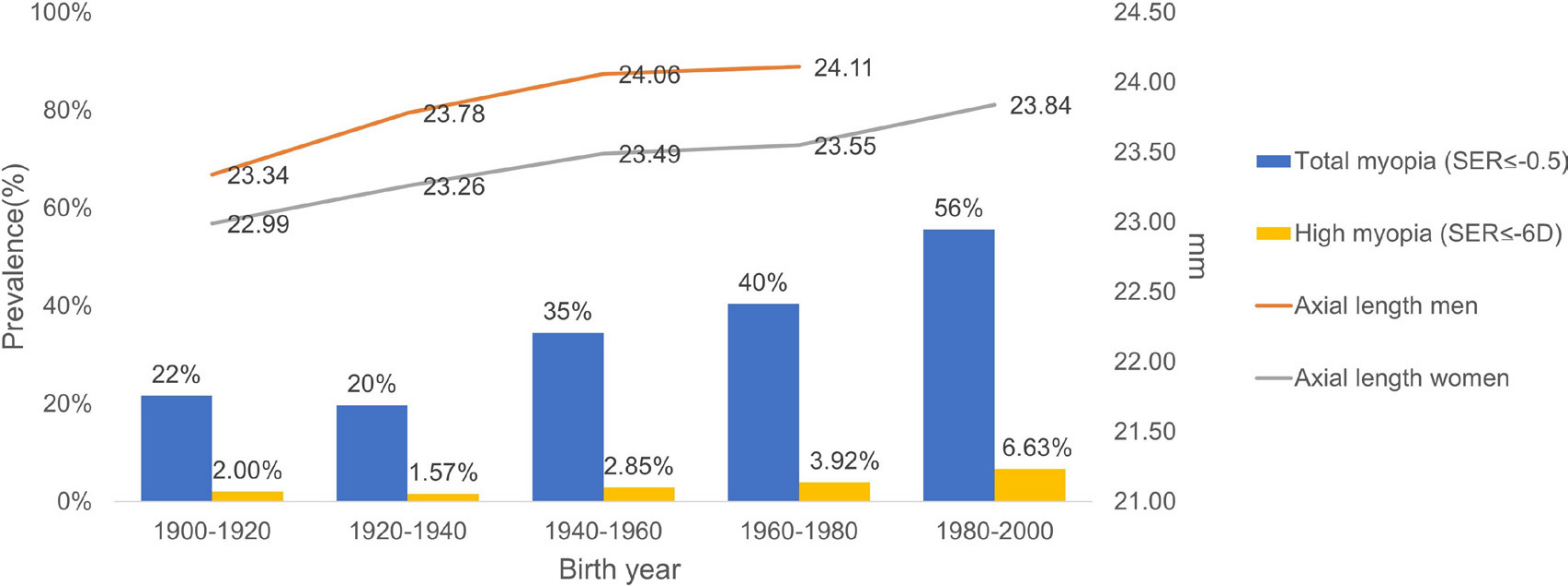
Prevalence of myopia (spherical equivalent refraction (SER) ≤–0.5 D; blue bars) and high myopia (SER ≤–6.0 D; yellow bars), and mean axial length in millimeters (right y-axis) across birth years.

The myopia prevalence increase in 20-year time periods from 1900 to 2000 is shown in figure 1. The prevalence increased from 22% to 56% in this century, an increase of 2.5 times. The prevalence of high myopia increased from 2.00% in 1900–1920 to 6.63% in 1980–2000, an increase of 3.3 times (figure 1). When performing logistic regression corrected for sex, the OR for each time period compared with 1900–1920 rose, after initial stabilisation, to an even higher level: 1920–1940, OR 0.88 (95% CI 0.74 to 1.04); 1940–1960, OR 1.90 (95% CI 1.60 to 2.25); 1960–1980, OR 2.43 (95% CI 2.02 to 2.92); 1980–2000, OR 4.79 (95% CI 3.72 to 6.18). Similar results were found with high myopia 1920–1940, OR 0.79 (95% CI 0.48 to 1.37); 1940–1960, OR 1.45 (95% CI 0.90 to 2.47); 1960–1980, OR 1.99 (95% CI 1.21 to 3.46); 1980–2000, OR 3.30 (95% CI 1.79 to 6.21).

### Temporal trend of AL and SE

**Figure 2:**
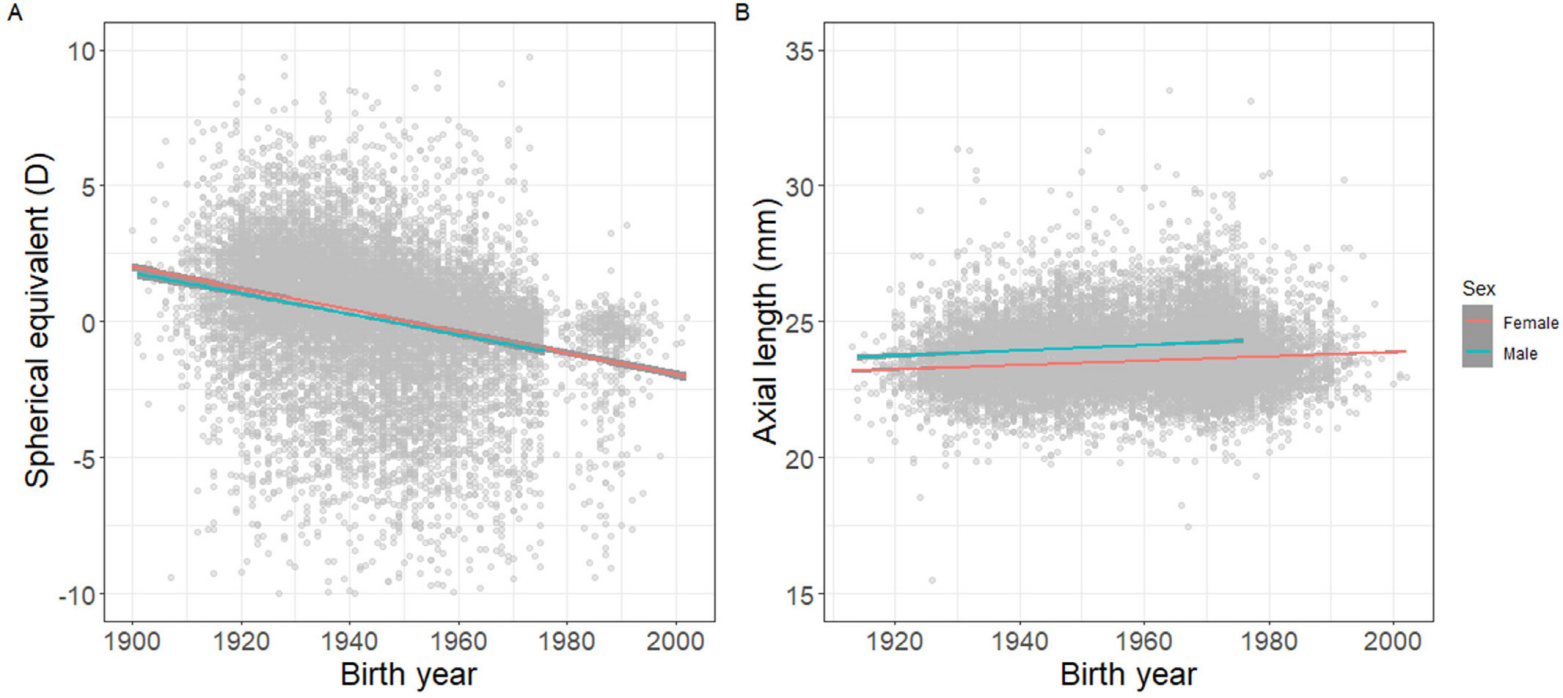
Spherical equivalent (**A**) and axial length (**B**) by birth year. Curves represent linear regression lines, with 95% CIs shaded in grey. Red lines indicate males; blue lines indicate females.

The mean AL increased by +0.77 mm (p<0.001) for men and by +0.85 mm (p<0.001) for women (table 1 and figure 1). Correspondingly, SE decreased by −2.7 D for women from 1900 to 2000 (p<0.001) and by −1.3 D for men from 1900 to 1980 (p<0.001). When stratified by sex, a similar trend was observed for both men and women in SE and AL (figure 2A,B). Using a linear model, AL was found to be significantly higher for each birth year group when compared with 1900–1920, except for 1920–1940 (1920–1940, β 0.28 mm (95% CI −0.04 to 0.59); 1940–1960, β 0.48 mm (95% CI 0.16 to 0.80); 1960–1980, β 0.56 mm (95% CI 0.24 to 0.87); 1980–2000, β 0.53 mm (95% CI 0.20 to 0.85)). SE showed a corresponding trend over time when compared with 1900–1920 (1920–1940, β −0.17 D (95% CI −0.35 to 0.01); 1940–1960, β −1.20 D (95% CI −1.37 to −1.02); 1960–1980, β −1.61 D (95% CI −1.81 to −1.41); 1980–2000, β −2.58 D (95% CI −2.88 to −2.27)). No significant interactions were found between sex and birth year or height, indicating that effect modification was unlikely.

### Role of education

We used a four-way decomposition approach to examine the relative contributions of mediation and moderation (interaction) by education on the association between birth year and myopia prevalence (online supplemental figure 2). We first compared the birth years 1920–1940 and 1960–1980, because both these strata had a large number of study participants and were significantly different in myopia prevalence (table 1 and online supplemental table 3). As demonstrated in online supplemental figure 2, participants born between 1960 and 1980 had a higher likelihood of having myopia (total excess relative risk (ERR 1.78). Accountable for this increase were the CDE (ERR 0.81 (95% CI 0.52 to 1.09)) of birth year on myopia prevalence, the INTref effect (ERR 0.74 (95% CI 0.39 to 1.11)), and the PIE (ERR 0.20 (95% CI 0.13 to 0.26)). The CDE accounted for 45% (95% CI 32% to 58%) of the total effect, the INTref effect contributed 41% (95% CI 24% to 61%) and the PIE contributed 11% (95% CI 7% to 15%). Comparisons between other birth year strata can be found in online supplemental table 4. Similar, though smaller effects were observed when comparing medium versus high and low versus medium education levels. Notably, a significant INTref effect was also detected when comparing low versus medium education levels (table 2).

**Table 2:** Four-way decomposition of mediation by education level on the effect of birth year on myopia prevalence

	Mediation analysis myopia			
	Excess relative risk	95% CI	Proportion†	95% CI
Low versus high				
Controlled direct effect	0.81**	0.52 to 1.09	0.45**	0.32 to 0.58
Reference interaction effect	0.74**	0.39 to 1.11	0.41**	0.24 to 0.61
Mediated interaction effect	0.04	−0.11 to 0.17	0.02	−0.06 to 0.09
Pure indirect effect	0.20**	0.13 to 0.26	0.11**	0.07 to 0.15
Total effect	1.78		1.0	
Medium versus high				
Controlled direct effect	0.61**	0.37 to 0.87	0.37*	0.27 to 0.45
Reference interaction effect	1.25**	0.64 to 2.08	0.75*	0.40 to 1.17
Mediated Interaction effect	−0.48	−1.12 to 0.07	−0.29	−0.68 to 0.04
Pure indirect effect	0.29**	0.15 to 0.44	0.17	0.09 to 0.29
Total effect	1.67		1.0	
Low versus medium				
Controlled direct effect	1.49**	1.02 to 1.98	0.88**	0.65 to 0.1.16
Reference interaction effect	0.52**	0.25 to 0.84	0.13**	0.16 to 0.44
Mediated interaction effect	−0.26*	−0.50 to −0.04	−0.16*	−0.29 to −0.03
Pure indirect effect	−0.07**	−0.11 to −0.04	−0.04**	−0.07 to −0.02
Total effect	1.68		1.0	

*p<0.001, **p<0.05.

†The proportion of the total effect should be interpreted with caution when not all relative risks are either positive or negative. Mixed signs in relative risks indicate opposite directions of effects, complicating the interpretation and potentially leading to unreliable conclusions.

* *p<0.001, **p<0.05.

† The proportion of the total effect should be interpreted with caution when not all relative risks are either positive or negative. Mixed signs in relative risks indicate opposite directions of effects, complicating the interpretation and potentially leading to unreliable conclusions.

### Estimated visual impairment due to myopia

**Figure 3:**
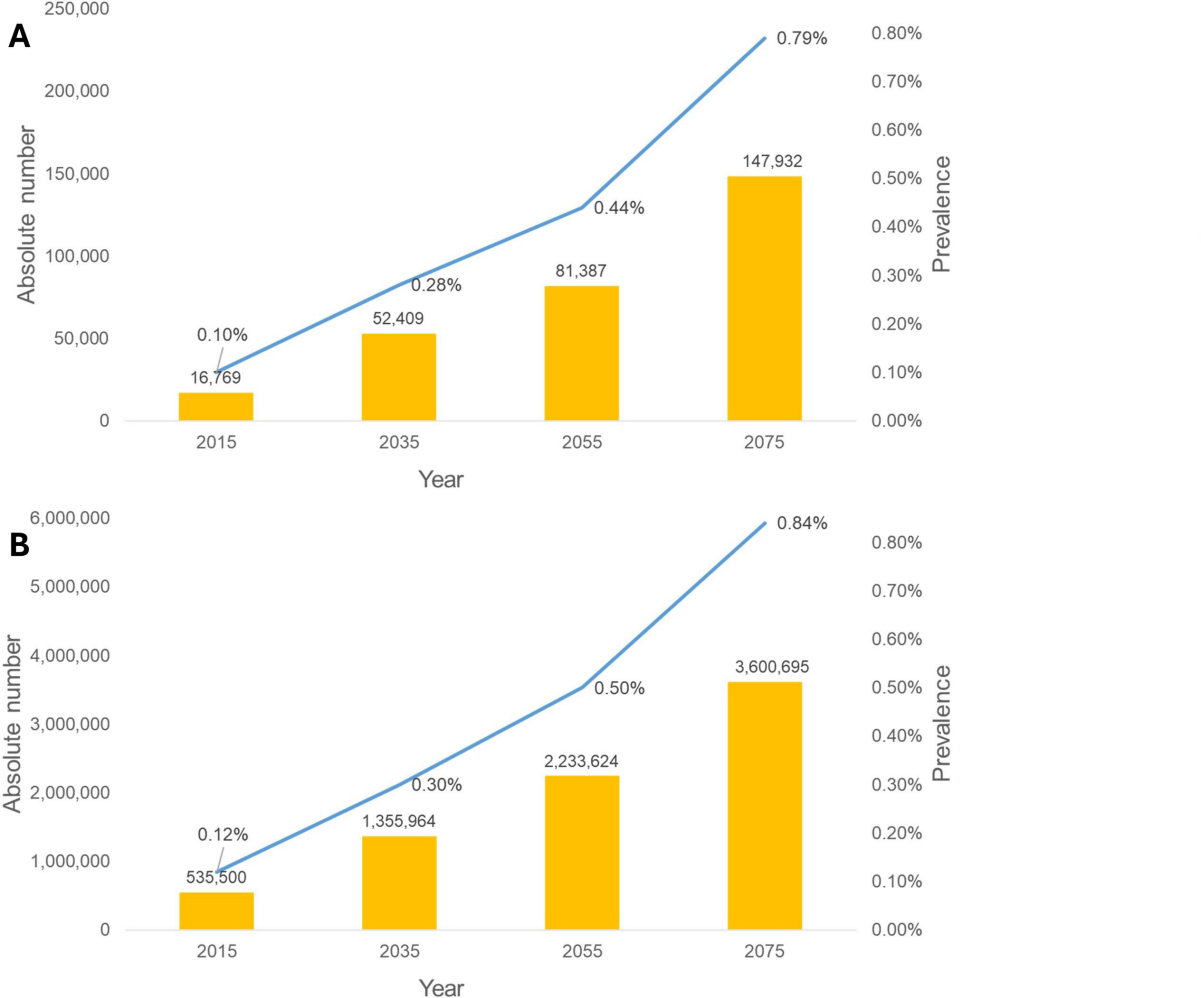
Visual impairment due to myopia in the Netherlands (**A**) and in Europe (**B**). Yellow bars depict the absolute number of visually impaired, the blue line depicts the prevalence (%) of visual impairment due to myopia.

We estimated that in total 1 856 707 Dutch aged 75 and older will be myopic in 2075, of whom 219 821 will be highly myopic (online supplemental table 5). Building on the risk analyses by Tideman *et al*,^11^ this translates to 147 932 individuals who are expected to become visually impaired due to myopia-related complications in 2075. This would constitute 0.79% of the Dutch population. Compared to 2015, this would be an eight times increase within 60 years in prevalence (0.10%–0.80%) (online supplemental table 4 and figure 3A). Similarly, for Europe, we anticipate an increase from approximately 530 600 visually impaired individuals in 2015 to 3.6 million in 2075 due to myopia-related complications. Consequently, the prevalence of visual impairment due to myopia in Europe is expected to rise from 0.12% in 2015 to 0.84% in 2075. (figure 3B)

## Discussion

Our study, including data from an entire century, revealed a consistent increase in myopia prevalence and AL in the Netherlands. From 1900 to 2000, the prevalence of myopia increased by a factor of 2.5; the prevalence of high myopia increased even by 3.3 times. The mean AL increased by +0.77 mm in men within 80 years and by +0.85 mm in women within 100 years. Correspondingly, the mean SE decreased by −1.3 D for men (1900–1980) and −2.7 D for women (1900–2000). Extrapolating these data and the reported risks of visual impairment, we predict that the prevalence of low vision and blindness due to myopia will increase eightfold by the year 2075.^22 23^

Risk factors that have been associated with myopia are education, near work, urbanisation, socioeconomic status and time spent outdoors.^24^,^27^ Among these, educational attainment is among the oldest known risk factors for myopia, first described in 1886.^28^ Despite fundamental changes in educational systems over time, the association has remained remarkably consistent.^24^ This relationship is thought to be mediated by increased near work and reduced outdoor exposure, both of which are common features of academic life.^2^ Our data show a significant increase in education levels in the Netherlands over time (table 1), which coincided with an expansion in mandatory years of education: a mandatory education was stated to be 6 years in 1901, with 7 years in 1928, with 8 years in 1942 and with 9 years in 1969, when education became compulsory up to age 16 years.^29^ Using the four-way decomposition approach, we studied the causal role of education on the myopia increase. Our results suggest that approximately 55% of the increase in prevalence is somewhat affected by education, however, it had a relatively small direct effect. The majority of its contribution was through interaction with birth year, indicating that education plays a role in how other generational factors influence myopia prevalence. Notably, the effect of birth year on myopia prevalence independent of education was also considerable. We interpret birth year as a proxy for unmeasured societal and behavioural risk factors such as urbanisation, increased near work and reduced outdoor time. In the Netherlands, urbanisation increased from 37% of the population in 1850 to 60% in 1960, and 93% in 2023.^30 31^ While these shifts likely coincided with a decline in outdoor activity among children, detailed and reliable data on changes in childhood outdoor exposure or near work throughout the 20th century are lacking. Educational attainment, which increased substantially over time (table 1), may therefore partially reflect these cumulative lifestyle and environmental changes. Emerging evidence suggests that genetic and behavioural factors exert additive effects on myopia development, contributing to the rising prevalence.^32 33^ Future studies are needed to further explore the potential role of gene–environment interactions in driving intergenerational changes in myopia risk. Our findings align with studies conducted in Europe over shorter periods. In the UK, myopia prevalence increased from 20% for those born between 1939 and 1944 to 30% for those born between 1965 and 1970.^34^ We also found 20% prevalence for 1920–1940 and 40% for 1960–1980. The extra decade in the second time period may be held accountable for the higher frequency. A meta-analysis of European cohort studies reported in 2010 found a myopia prevalence slightly lower than ours for the youngest generation.^6^ At that time, a 47.2% prevalence was found for 25–29 year-olds, while our report 14 years later found a 56% myopia prevalence in women of this age. Although it is likely that the higher prevalence is explained by the generation effect, the slight difference in myopia definition (−0.75D rather than −0.5 D)^20^ may have contributed to this difference.

Based on our data, we predict that by 2075, 0.8% of the population will become visually impaired due to myopia. In 2007 the total prevalence of visual impairment in the Netherlands was estimated at 1.8%; this was estimated to increase to 2.0% by 2020.^35^ In Western European countries, a similar increase was found with the prevalence of moderate to severe visual impairment increasing by 0.2% between 2010 and 2020 (3.30–3.53%), the prevalence of blindness showed a smaller increase of 0.02% (0.32–0.35%).^22^ The demographics of the Dutch population make it highly comparable to other high-income Western European countries.^36 37^ The leading causes of visual impairment in Western European countries are cataract (0.35%), age-related macular degeneration (0.24%), diabetic retinopathy (0.08%) and glaucoma (0.08%).^38^ Interestingly, as early as 1998, myopic retinal complications were identified as the most significant cause of visual impairment before age 75.^23^ Our predictions suggest that by 2070, the rate of visual impairment due to myopia will have increased eightfold compared with its prevalence in the previous century (figure 3).^138^,^40^

Visual impairment not only affects the lives of patients, but it also has an enormous impact on economic productivity.^41^ The costs related to visual complications caused by myopia were estimated at US$250 billion annually.^42^ Moreover, myopia is associated with a lower quality of life, which affects productivity, mobility and daily activities, leading to even higher, often unquantifiable costs.^42 43^ Our findings underscore the urgent need for long-term healthcare planning, as the projected increase in myopia-related complications is expected to place substantial strain on already burdened eye care services.^38^ Preventive strategies and early interventions could play an important role in reducing the future burden of vision loss in ageing populations. Visual impairment has profound effects on quality of life at all ages, contributing to higher rates of unemployment, depression, anxiety, social isolation, increased risk of falls and fractures, and earlier admission to nursing homes.^44^ Even when potential complications are disregarded, uncorrected myopia remains the leading cause of visual impairment worldwide, with an estimated annual productivity loss of US$244 billion.^43^ Southeast Asia, South Asia and East Asia stand out as having well over twice the burden of any other region as a percentage of GDP: 1.35%, 1.30% and 1.27%, respectively.^43^

Policy makers in government as well as the private sectors should become aware of this expected decrease in productivity due to myopia. Strategies aimed at addressing myopia undeniably hold significant economic value for society.^43^ Evidence-based strategies for myopia control include increasing time spent outdoors, early identification of at-risk children, school-based education campaigns, and the use of optical and pharmacological interventions.^45 46^

### Strengths and limitations

Strengths of this study were: (1) the large data set from participants born during a period of 100 years, all from the same source population in the Netherlands. The individual-level data allowed us to accurately perform in-depth analyses on SE, AL and covariates, a clear advantage over meta-analyses relying on summary statistics from different populations.^47^ Although cycloplegic refraction was not used, the inclusion of adults aged ≥24 years minimises the risk of accommodative bias.^48^ Additionally, while different biometry instruments were used across cohorts, the devices applied in this study (Lenstar and IOLMaster) have been shown to yield highly comparable AL measurements.^49^ ^50 51^ (2) In addition to education, the Netherlands shares many social, economic and healthcare characteristics with high-income nations such as Germany, France and the Scandinavian countries. The demographic characteristics of the Dutch population place our study in the European mainstream.^36 37^ This alignment strongly supports the validity of extrapolating our data to the entire region.^36 37^ (3) An objective and consistent methodology was used to diagnose myopia across generations. The inclusion of AL strengthens our data as a robust starting point for evaluating myopia progression in future generations. This will aid in predicting myopic complications and their impact on society.^2^

However, our study also has limitations. One of the limitations of cohort studies is the potential for selection bias, particularly the ‘healthy cohort bias’. It is generally assumed that healthier, wealthier and more highly educated individuals are more likely to participate in such studies. This may have led to overestimation of the prevalence of myopia. However, as described earlier, the educational level of our study population closely matched that reported in other Western European countries, thereby reducing the likelihood of overestimation. Also, some covariates were collected through questionnaires, which may have introduced self-report bias. Nonetheless, key variables such as education, which were crucial for our mediation analysis, have been shown to be reliable even when self-reported.^52^,^56^ Additionally, the majority of participants born between 1960 and 2000 were female, and the youngest generation of adults were the mothers from our Generation R study. Previous studies have mostly reported a gender difference for older generations, and we did not find a significant gender difference in myopia. Hence, this is unlikely to have distorted our findings. Another potential limitation is that we excluded all pseudophakic participants and those with suspected cataract when estimating myopia prevalence to minimise misclassification bias. Nonetheless, residual bias may persist if unrecognised lens changes or other ocular comorbidities influenced refractive status. Moreover, as myopia is associated with an increased HR of cataract, this may have led to an under-representation of myopia prevalence, especially in the older generations, as more of the myopic participants have undergone cataract extraction.^2^ We believe this distortion is limited, as the AL data from all participants show a similar increasing trend of myopic elongation across generations. Lastly, in estimating visual impairment caused by myopia, several assumptions were made. We assumed that reported risk levels remain constant over time and across populations; we did not account for changes in healthcare access, treatment strategies or public health interventions. Additionally, we assumed that the prevalence rate in Rotterdam accurately represents the prevalence rate in the Netherlands and that myopia does not affect mortality before the age of 75. Although one study reported a hazard relative risk of 1.35 for mortality in myopes, it lacked adjustment for lifestyle factors and has not been replicated.^57^ Nevertheless, these assumptions may lead to a slight over- or underestimation of the true burden of visual impairment.

## Conclusion

Our population-based studies revealed a significant rise in myopia prevalence over the past 100 years in a Western European country. The prevalence of myopia and high myopia increased by factors of 2.5 and 3.5, respectively, a rise which was partly driven by education. Mean AL increased by 0.8 mm in men and 0.6 mm in women, while mean SE decreased by −2.5 D. With a prevalence exceeding 50% among young adults, myopia has reached epidemic proportions. As the young generations age, the impact on society and healthcare will intensify. We estimate that by 2035, myopic complications will become the leading cause of irreversible visual impairment in Western Europe, highlighting the need for myopia prevention strategies.

## Supplementary material

10.1136/bmjph-2024-002307 online supplemental figure 1

10.1136/bmjph-2024-002307 online supplemental figure 2

10.1136/bmjph-2024-002307 online supplemental file 1

## Data Availability

Data are available upon reasonable request.

## References

[1] Flaxman SR, Bourne RRA, Resnikoff S (2017). Global causes of blindness and distance vision impairment 1990–2020: a systematic review and meta-analysis. Lancet Glob Health.

[2] Haarman AEG, Enthoven CA, Tideman JWL (2020). The Complications of Myopia: A Review and Meta-Analysis. Invest Ophthalmol Vis Sci.

[3] Bullimore MA, Brennan NA (2023). The underestimated role of myopia in uncorrectable visual impairment in the United States. Sci Rep.

[4] Holden BA, Fricke TR, Wilson DA (2016). Global Prevalence of Myopia and High Myopia and Temporal Trends from 2000 through 2050. Ophthalmology.

[5] Pan C-W, Ramamurthy D, Saw S-M (2012). Worldwide prevalence and risk factors for myopia. Ophthalmic Physiol Opt.

[6] Williams KM, Verhoeven VJM, Cumberland P (2015). Prevalence of refractive error in Europe: the European Eye Epidemiology (E(3)) Consortium. Eur J Epidemiol.

[7] Matsumura S, Ching-Yu C, Saw S-M, Ang M, Wong TY (2020). Updates on Myopia: A Clinical Perspective.

[8] Wolfram C, Höhn R, Kottler U (2014). Prevalence of refractive errors in the European adult population: the Gutenberg Health Study (GHS). Br J Ophthalmol.

[9] Anastasopoulos E, Haidich AB, Coleman AL (2018). Risk factors for Age-related Macular Degeneration in a Greek population: The Thessaloniki Eye Study. Ophthalmic Epidemiol.

[10] Theophanous C, Modjtahedi BS, Batech M (2018). Myopia prevalence and risk factors in children. Clin Ophthalmol.

[11] Tideman JWL, Snabel MCC, Tedja MS (2016). Association of Axial Length With Risk of Uncorrectable Visual Impairment for Europeans With Myopia. JAMA Ophthalmol.

[12] (2013). Myopia Stabilization and Associated Factors Among Participants in the Correction of Myopia Evaluation Trial (COMET). Invest Ophthalmol Vis Sci.

[13] Lee SS-Y, Lingham G, Sanfilippo PG (2022). Incidence and Progression of Myopia in Early Adulthood. JAMA Ophthalmol.

[14] Jaddoe VWV, van Duijn CM, Franco OH (2012). The Generation R Study: design and cohort update 2012. Eur J Epidemiol.

[15] Kooijman MN, Kruithof CJ, van Duijn CM (2016). The Generation R Study: design and cohort update 2017. Eur J Epidemiol.

[16] Ikram MA, Brusselle G, Ghanbari M (2020). Objectives, design and main findings until 2020 from the Rotterdam Study. Eur J Epidemiol.

[17] Statistics UIf (2012). International Standard Classification of Education: ISCED 2011.

[18] VanderWeele TJ (2014). A unification of mediation and interaction: a 4-way decomposition. Epidemiology (Sunnyvale).

[19] Yung Y-F, Lamm M, Zhang W Causal mediation analysis with the causalmed procedure.

[20] CBS (2021). Bevolkingspiramide.

[21] Eurostat (2023). Eurostat.

[22] model VG (2020). GBD Region Map & Estimates of Vision Loss Western Europe.

[23] Klaver CCW (1998). Age-Specific Prevalence and Causes of Blindness and Visual Impairment in an Older Population. Arch Ophthalmol.

[24] Williams KM, Bertelsen G, Cumberland P (2015). Increasing Prevalence of Myopia in Europe and the Impact of Education. Ophthalmology.

[25] Verhoeven VJM, Buitendijk GHS, Consortium for Refractive Error and Myopia (CREAM) (2013). Education influences the role of genetics in myopia. Eur J Epidemiol.

[26] Dutheil F, Oueslati T, Delamarre L (2023). Myopia and Near Work: A Systematic Review and Meta-Analysis. Int J Environ Res Public Health.

[27] Morgan IG, Wu P-C, Ostrin LA (2021). IMI Risk Factors for Myopia. Invest Ophthalmol Vis Sci.

[28] Cohn HL, Turnbull WP (1886).

[29] Ministerie van Onderwijs CeW (1969). Leerplichtwet 1969.

[30] Paping R (2014). General dutch population development 1400-1850: cities and countryside.

[31] United Nations Department of E, Social A (2019). World Urbanization Prospects: The 2018 Revision: United Nations.

[32] Enthoven CA, Tideman JWL, Polling JR (2019). Interaction between lifestyle and genetic susceptibility in myopia: the Generation R study. Eur J Epidemiol.

[33] Yu M, Hu Y, Han M (2023). Global risk factor analysis of myopia onset in children: A systematic review and meta-analysis. PLoS ONE.

[34] Cumberland PM, Bountziouka V, Hammond CJ (2022). Temporal trends in frequency, type and severity of myopia and associations with key environmental risk factors in the UK: Findings from the UK Biobank Study. PLoS ONE.

[35] Limburg H (2007). Epidemiologie van visuele beperkingen en een demografische verkenning.

[36] Rozendal P, Moors H (1983). Attitudes towards population trends and population policy in the Netherlands, compared with some data from other Western European countries. Europ Demogr Inf Bull.

[37] Coleman D, Garssen J (2002). The Netherlands:Paradigm or Exception in Western Europe’s Demography?. DemRes.

[38] Blindness GBD, Vision Impairment C, Vision Loss Expert Group of the Global Burden of Disease S (2021). Causes of blindness and vision impairment in 2020 and trends over 30 years, and prevalence of avoidable blindness in relation to VISION 2020: the Right to Sight: an analysis for the Global Burden of Disease Study. Lancet Glob Health.

[39] Deng Y, Qiao L, Du M (2022). Age-related macular degeneration: Epidemiology, genetics, pathophysiology, diagnosis, and targeted therapy. Genes Dis.

[40] Holden BA, Wilson DA, Jong M (2015). Myopia: a growing global problem with sight-threatening complications. Community Eye Health.

[41] Frick KD, Foster A (2003). The magnitude and cost of global blindness: an increasing problem that can be alleviated. Am J Ophthalmol.

[42] Chua SYL, Foster PJ, Ang M, Wong TY (2020). Updates on Myopia: A Clinical Perspective.

[43] Naidoo KS, Fricke TR, Frick KD (2019). Potential Lost Productivity Resulting from the Global Burden of Myopia: Systematic Review, Meta-analysis, and Modeling. Ophthalmology.

[44] World Health Organization (2023). Blindness and vision impairment.

[45] Yam JC, Zhang XJ, Zhang Y (2023). Effect of Low-Concentration Atropine Eyedrops vs Placebo on Myopia Incidence in Children: The LAMP2 Randomized Clinical Trial. JAMA.

[46] He X, Sankaridurg P, Wang J (2022). Time Outdoors in Reducing Myopia: A School-Based Cluster Randomized Trial with Objective Monitoring of Outdoor Time and Light Intensity. Ophthalmology.

[47] Bullimore MA, Lee SS-Y, Schmid KL (2023). IMI-Onset and Progression of Myopia in Young Adults. Invest Ophthalmol Vis Sci.

[48] Sanfilippo PG, Chu B-S, Bigault O (2014). What is the appropriate age cut-off for cycloplegia in refraction?. Acta Ophthalmol.

[49] Huang J, McAlinden C, Huang Y (2017). Meta-analysis of optical low-coherence reflectometry versus partial coherence interferometry biometry. Sci Rep.

[50] Francis van der Mooren RdV (2022). Steeds Meer Hoogopgeleiden in Nederland: Wat Voor Beroep Hebben Ze?.

[51] Bertoletti A (2023). Forecasting progress towards the eu-level targets of the european education area.

[52] Vo CQ, Samuelsen P-J, Sommerseth HL (2023). Validity of self-reported educational level in the Tromsø Study. Scand J Public Health.

[53] Centraal Bureau voor de Statistiek DHHB (2022). Andries de jong (pbl) strc, corina huisman (cbs), coen van duin, (cbs) thp, lenny stoeldraijer (cbs) regionale bevolkingsen huishoudens prognose 2022–2050; steden en randgemeenten groeien verder.

[54] Li X, Li L, Qin W (2023). Urban Living Environment and Myopia in Children. JAMA Netw Open.

[55] Czepita D, Mojsa A, Zejmo M (2008). Prevalence of myopia and hyperopia among urban and rural schoolchildren in Poland. Ann Acad Med Stetin.

[56] United Nations DoEaSA (2018). World Urbanization Prospects: The 2018 Revision.

[57] Wang JJ, Mitchell P, Simpson JM (2001). Visual impairment, age-related cataract, and mortality. Arch Ophthalmol.

