# Supplementary figures and images for: Trends in myopia prevalence and projected visual impairment in Western Europe: a pooled analysis of Dutch population-based cohorts (1900–2000)
Source: BMJ Public Health. 2025 Sep 29;3(2):e002307. doi: 10.1136/bmjph-2024-002307 (PMC12481288; doi:10.1136/bmjph-2024-002307)

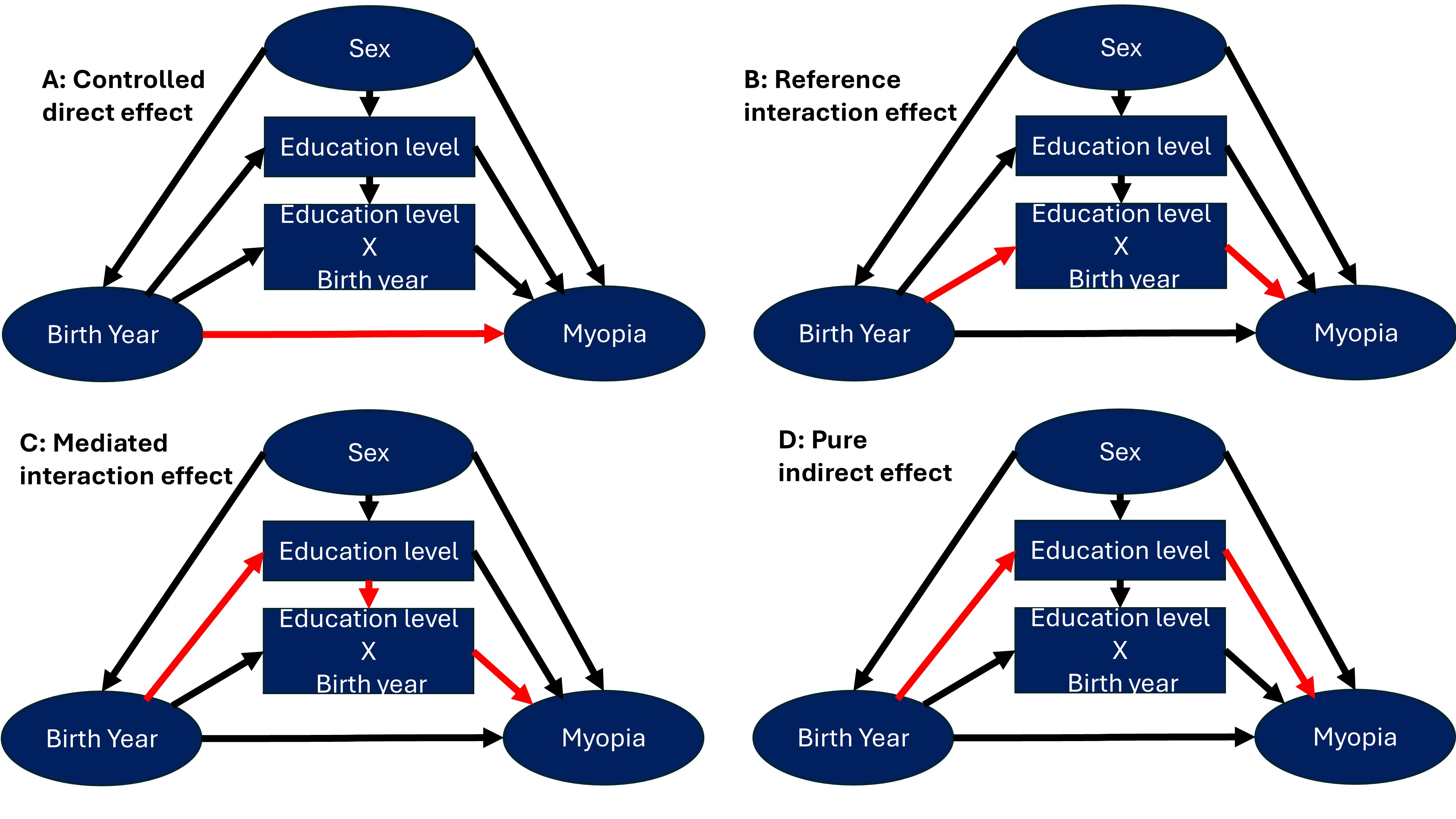

Supplement: online supplemental figure 1 [file bmjph-3-2-s001.jpg]

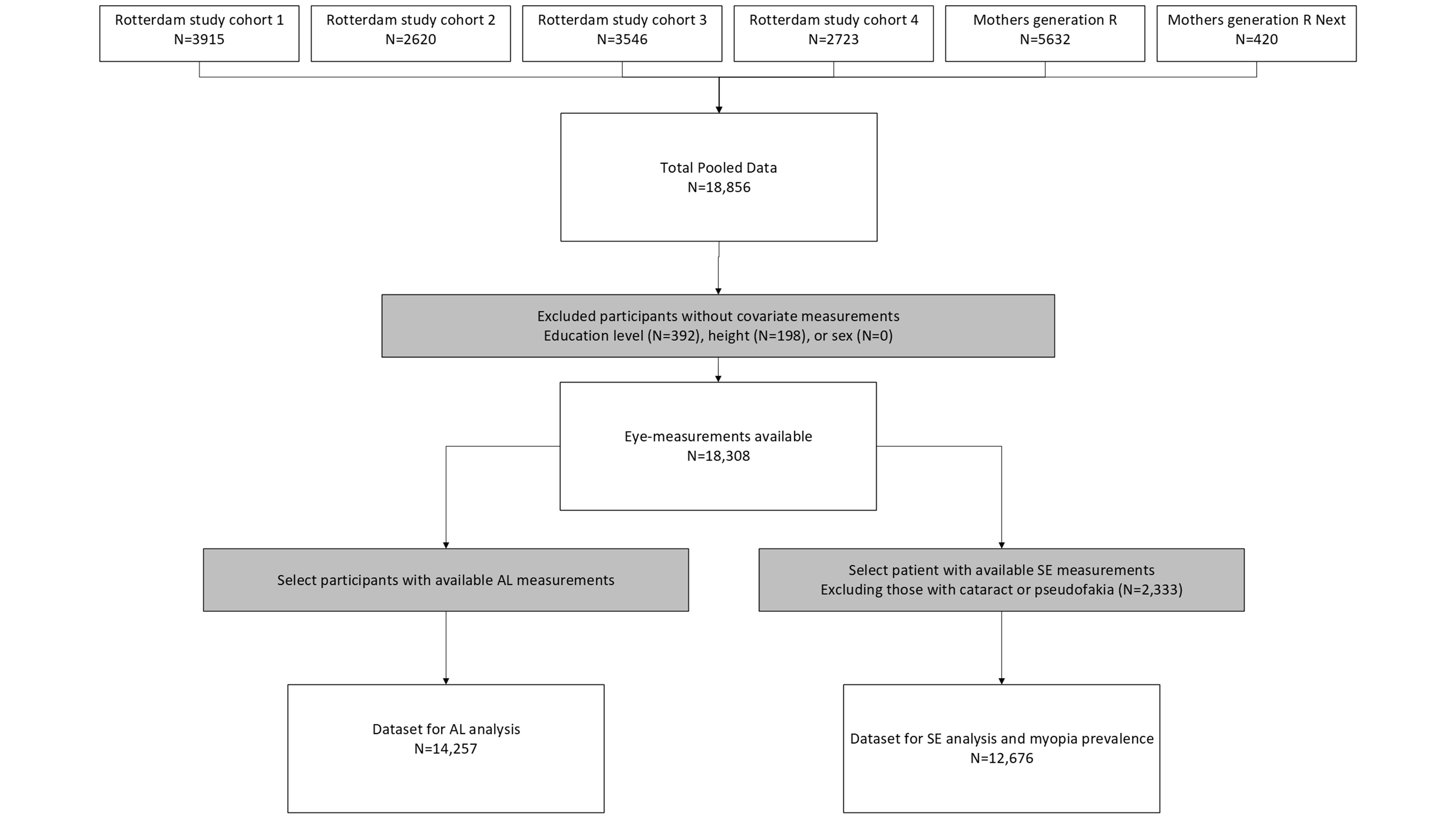

Supplement: online supplemental figure 2 [file bmjph-3-2-s002.jpg]
