# Supplementary material for: Trends in myopia prevalence and projected visual impairment in Western Europe: a pooled analysis of Dutch population-based cohorts (1900–2000)
Source: BMJ Public Health. 2025 Sep 29;3(2):e002307. doi: 10.1136/bmjph-2024-002307 (PMC12481288; doi:10.1136/bmjph-2024-002307)
Supplement: online supplemental file 1 [file bmjph-3-2-s003.docx]

# Supplements

# Supplementary figures:

# *Supplementary Figure 1: Flow chart describing data selection process.*

*Supplementary Figure 2: Directed Acyclic Graph of the mediation analysis to evaluate the mediating and moderating effects of education on the association between birth year and myopia. Sex may act as a confounder. Red arrow indicates each way of the decomposition: A: Controlled direct effect, B: Reference interaction effect, C: Mediated interaction effect, D: Pure indirect effect.*

# Supplementary Tables:

| **Demographics by study cohort** | | | | | | | |  |
| --- | --- | --- | --- | --- | --- | --- | --- | --- |
|  | **RSI** | **RSII** | **RSIII** | **RSIV** | **Generation R (mothers)** | **Generation R Next (mothers)** | **P-value*** |  |
| Participants | 3873 | 2565 | 3530 | 2707 | 5237 | 419 |  | |
| Birth year(Range) | 1901-1938 | 1905-1945 | 1919-1960 | 1920-1976 | 1957-1989 | 1975-2000 |  | |
| Mean Age (SD)* | 65.3 (±6.6) | 64.1(±7.4) | 56.7 (±6.6) | 56.7 (±10.9) | 37.1 (±5.0) | 34.4 (±4.4) | **<0.001** | |
| Sex (% female) | 2252(57.6%) | 1438(54.9%) | 2002(56.5%) | 1550(56.9%) | 5456(100%) | 434(100%) |  | |
| Education level (%) |  |  |  |  |  |  | **<0.001** | |
| Low | 2279(58.3%) | 1371 (52.3) | 1621 (45.8) | 1650 (60.6) | 82 (4.3%) | 1 (0.2%) |  | |
| Medium | 1174 (30.0%) | 750 (28.6%) | 968 (27.3%) | 206 (7.6%) | 1165 (61.4%) | 79 (18.2%) |  | |
| High | 420 (10.7%) | 444 (17.0%) | 941 (26.6%) | 851 (31.3%) | 650 (34.3%) | 344 (79.3%) |  | |
| Missing | 38(1.0%) | 54(2.1%) | 13(0.4%) | 16(0.6%) | 133(2.4%) | 10(2.3%) |  | |
| AL (mm) | 23.5 (±1.2) | 23.5 (±1.2) | 23.8 (±1.3) | 23.8 (±1.3) | 23.7(±1.0) | 23.8 (±1.2) |  | |
| Male | 23.9(±1.2) | 23.9(±1.2) | 24.1(±1.3) | 24.1(±1.3) | NA | NA |  | |
| Female | 23.2(±1.2) | 23.3(±1.1) | 23.5(±1.2) | 23.5(±1.2) | 23.7(±1.2) | 23.8(±1.2) |  | |
| Height (cm) |  |  |  |  |  |  |  | |
| Male | 175.6(±6.7) | 175.6(±6.6) | 178.3(±7.0) | 179.7(±7.8) | NA | NA |  | |
| Female | 162.4(±6.3) | 162.6(±6.2) | 164.4(±6.4) | 165.7(±7.5) | 167.6(±7.4) | 168.8(±6.6) |  | |
| SE(D) | 0.9 (±2.5) | 0.5 (±2.5) | -0.3 (±2.6) | -0.3 (±2.5) | NA | -1.5 (±2.4) |  | |
| *Age at examination. | | | | | | | |  |

supplementary table 1. Demographics stratified per cohort

|  | **1900-1920** | **1920-1940** | **1940-1960** | **1960-1980** | **1980-2000** |
| --- | --- | --- | --- | --- | --- |
| Rotterdam Study I | 801 | 3110 | 0 | 0 | 0 |
| Rotterdam Study II | 116 | 1616 | 887 | 0 | 0 |
| Rotterdam Study III | 4 | 215 | 3324 | 0 | 0 |
| Rotterdam Study IV | 1 | 196 | 660 | 1866 | 0 |
| Generation R next (mothers) | 0 | 0 | 0 | 13 | 421 |
| Generation R (mothers) | 0 | 0 | 13 | 4810 | 633 |

Supplementary table 2. Distribution of cohort studies by birth years.

| ***Ocular measures by education level*** | | | | | | | | | | | |
| --- | --- | --- | --- | --- | --- | --- | --- | --- | --- | --- | --- |
|  | **1900-1920** | **N** | **1920-1940** | **N** | **1940-1960** | **N** | **1960-1980** | **N** | **1980-2000** | **N** |  |
| ***SE (D)*** |  |  |  |  |  |  |  |  |  |  |  |
| *Low educated* | 1.14(±2.64) | 580 | 1.03(±2.30) | 2830 | 0.15(±2.46) | 2266 | -0.36(±2.36) | 980 | -0.75 | 1 |  |
| *Medium educated* | 0.90(±2.43)* | 241 | 0.67(±2.58)* | 1455 | -0.27(±2.57)* | 1203 | -1.14(±2.55)* | 146 | -1.39(±2.43) | 76 |  |
| *High educated* | 0.31(±2.63)* | 77 | 0.23(±2.54)* | 605 | -0.79(±2.65)* | 1233 | -0.85(±2.43)* | 661 | -1.51(±2.44) | 329 |  |
| ***AL men (mm)*** |  |  |  |  |  |  |  |  |  |  |  |
| *Low educated* | 23.11(±0.89) | 8 | 23.73(±1.06) | 418 | 23.90(±1.38) | 636 | 24.02(±1.22) | 422 | Na | 0 |  |
| *Medium educated* | 23.58(±1.30) | 6 | 23.80(±1.22) | 411 | 23.99(±1.27) | 549 | 24.22(±1.05) | 57 | Na | 0 |  |
| *High educated* | 23.88(±0.68) | 4 | 24.01(±1.22)* | 263 | 24.34(±1.36)* | 610 | 24.33(±1.30)* | 297 | Na | 0 |  |
| ***AL women (mm)*** |  |  |  |  |  |  |  |  |  |  |  |
| *Low educated* | 22.94(±1.19) | 27 | 23.17(±1.11) | 988 | 23.32(±1.17) | 1308 | 23.35(±1.22) | 845 | 23.26(±0.81) | 84 |  |
| *Medium educated* | 22.78(±1.54) | 11 | 23.42(±1.27)* | 342 | 23.61(±1.24)* | 483 | 23.55(±1.19)* | 1938 | 23.46(±1.21) | 508 |  |
| *High educated* | 24.17(±0.53) | 2 | 23.54(±1.15)* | 115 | 23.83(±1.29)* | 497 | 23.85(±1.25)* | 3003 | 23.85(±1.26)* | 387 |  |
| * Significantly different from low educated, using Tukey HSD comparison.  Supplementary table 3 : Distribution of ocular measures over education levels over time.   \| Year \| Population 75+ years^a^ \| Number of myopes 75+ years^b^ \| Number of high myopes 75+ years^c^ \| Visual impairement  (absolute) \| Visual impairment (Prevalence %)^e^ \| \| --- \| --- \| --- \| --- \| --- \| --- \| \| Netherlands \|  \|  \|  \|  \|  \| \| 2015 \| 1,277,510 \| 235,445 \| 20,057 \| 16,769 \| 0.10% \| \| 2035 \| 2,246,249 \| 722,169 \| 64,018 \| 52,409 \| 0.28% \| \| 2055 \| 2,806,603 \| 1,012,622 \| 110,019 \| 81,387 \| 0.44% \| \| 2075 \| 3,315,549 \| 1,636,886 \| 219,821 \| 147,932 \| 0.79% \| \| Europe \|  \|  \|  \|  \|  \| \| 2015 \| 40,795,625 \| 7,518,634 \| 640,491 \| 535,500 \| 0.12% \| \| 2035 \| 58,116,082 \| 18,684,320 \| 1,656,308 \| 1,355,964 \| 0.30% \| \| 2055 \| 77,025,780 \| 27,790,901 \| 3,019,411 \| 2,233,624 \| 0.50% \| \| 2075 \| 80,701,230 \| 39,842,197 \| 5,350,492 \| 3,600,695 \| 0.84% \| \| ^a^ predicted total population aged 75-95.  ^b^ (birth year prevalence myopia- birth year prevalence high myopia)*population 75+  ^c^ birth year prevalence high myopia*population 75+  ^d^ number of low myopes 75+*0.038+number of high myopes 75+*0.39  ^E^ absolute number of visually impaired / total population. \| \| \| \| \| \|   Supplementary table 4. Estimated number of visually impaired people in the Netherlands and Europe caused by myopia. | | | | | | | | | | | |

| **Low vs High education level** | | | | | | | |
| --- | --- | --- | --- | --- | --- | --- | --- |
| Periods compared | **Controlled direct effect** | **Reference interaction effect** | **Mediated Interaction effect** | **Pure indirect effect** | **Total effect** |  |  |
| 1900-1920 vs 1920-1940 | -0.17(-0.67-0.37) | 0.00(-0.50-0.47) | 0.00(-0.05-0.03) | 0.04(0.01-0.10)* | -0.14(0.71-1.09) |  |  |
| **Low vs High education level** | | | | | |  |  |
| 1900-1920 vs 1940-1960 | 0.56(0.02-1.14)* | 0.09(-0.35-0.58) | 0.11(-0.07-0.28) | 0.20(0.05-0.39)* | 0.96(1.61-2.40)** |  |  |
| 1900-1920 vs 1960-1980 | 0.55(0.03-1.20)* | 0.62(0.09-1.23)* | 0.01(-0.24-0.26) | 0.24(0.05-0.47)* | 1.42(1.98-3.05)** |  |  |
| 1900-1920 vs 1980-2000^a^ | 1.02(0.41-1.70)* | 64.8(0.02-91.59)* | -63.06(-89.51-1.92) | 0.91(0.24-1.89)* | 3.67(3.52-6.31)** |  |  |
| 1920-1940 vs 1940-1960 | 0.81(0.56-1.07)** | 0.16(-0.08-0.40) | 0.12(0.05-0.20)* | 0.15(0.11-0.21)** | 1.25(2.01-2.50)** |  |  |
| 1920-1940 vs 1960-1980 | 0.81(0.51-1.11)** | 0.74(0.38-1.09)** | 0.04(-0.09-0.16) | 0.21(0.13-0.27)** | 1.8(2.44-3.15)** |  |  |
| 1920-1940 vs 1980-2000^a^ | 1.32(0.97-1.72)** | 47.55(-0.21-56.71) | -45.39(-53.17-2.62) | 0.85(0.58-1.15)** | 4.33(4.22-6.69)** |  |  |
| 1940-1960 vs 1960-1980 | 0.00(-0.14-0.13) | 0.22(0.10-0.35)** | -0.01(-0.03-0.00)* | 0.03(0.01-0.06)** | 0.24(1.10-1.39)** |  |  |
| 1940-1960 vs 1980-2000^a^ | 0.28(0.12-0.47)* | 8.75(-0.14-10.47) | -8.25(-9.59-0.72) | 0.58(0.43-0.72)** | 1.36(1.89-2.99)** |  |  |
| 1960-1980 vs 1980-2000^a^ | 0.26(0.09-0.43)* | 5.71(-0.29-6.91) | -5.33(-6.29-0.73) | 0.26(0.13-0.41)** | 0.90(1.54-2.43)** |  |  |
| **intermediate vs High education level** | | | | | | | |
| 1900-1920 vs 1920-1940 | -0.13(-0.51-0.35) | 0(-0.39-0.39) | 0.00(-0.04-0.04) | 0.02(-0.01-0.08) | -0.11(0.69-1.18) | |  |
| 1900-1920 vs 1940-1960 | 0.45(0.00-0.96) | 0.24(-0.16-0.70) | 0.07(-0.15-0.26) | 0.14(-0.03-0.34) | 0.9(1.47-2.48)** | |  |
| 1900-1920 vs 1960-1980 | 0.43(-0.03-0.97) | 1.15(0.46-2.18)** | -0.54(-1.41-0.10) | 0.34(-0.01-0.82) | 1.38(1.81-3.28)** | |  |
| 1900-1920 vs 1980-2000 | 0.83(0.32-1.47)* | 1.98(0.76-3.83)* | -0.55(-2.05-0.61) | 0.33(-0.06-0.80) | 2.58(2.62-4.97)** | |  |
| 1920-1940 vs 1940-1960 | 0.63(0.42-0.86)** | 0.3(0.10-0.51)* | 0.09(0.01-0.18)* | 0.12(0.06-0.17)** | 1.14(1.88-2.43)** | |  |
| 1920-1940 vs 1960-1980 | 0.61(0.38-0.85)** | 1.26(0.67-2.10)** | -0.49(-1.15-0.02) | 0.29(0.16-0.43)** | 1.67(2.25-3.17)** | |  |
| 1920-1940 vs 1980-2000 | 1.04(0.76-1.37)** | 2.17(1.00-4.02)** | -0.48(-1.91-0.50) | 0.27(0.14-0.42)** | 2.99(3.25-4.96)** | |  |
| 1940-1960 vs 1960-1980 | -0.01(-0.13-0.11) | 0.31(0.13-0.56)* | -0.2( -0.38--0.06)* | 0.15(0.10-0.22)** | 0.25(1.07-1.48)* | |  |
| 1940-1960 vs 1980-2000 | 0.24(0.09-0.42)** | 0.69(0.30-1.21)* | -0.21(-0.56-0.07) | 0.14(0.09-0.21)** | 0.88(1.53-2.35)** | |  |
| 1960-1980 vs 1980-2000 | 0.22(0.07-0.37)* | 0.27(0.07-0.53)* | 0.00(-0.02-0.02) | 0.00(-0.01-0.01) | 0.49(1.17-1.88)** | |  |
| **Low vs Intermediate education level** | | | | | | | |
| 1900-1920 vs 1920-1940 | -0.11(-0.37-0.17) | -0.04(-0.27-0.20) | 0.00(-0.02-0.02) | 0.01(0.00-0.03) | -0.15(0.71-1.04) | |  |
| 1900-1920 vs 1940-1960 | 0.54(0.22-0.90)* | 0.15(-0.10-0.44) | 0.01(-0.01-0.04) | 0.02(-0.01-0.04) | 0.71(1.42-2.09)** | |  |
| 1900-1920 vs 1960-1980 | 1.2(0.69-1.78)** | 0.32(-0.03-0.68) | -0.18(-0.39--0.01)* | -0.05(-0.11-0.02) | 1.29(1.89-2.82)** | |  |
| 1900-1920 vs 1980-2000^a^ | 1.66(1.00-2.40)** | 24.59(0.76-35.92)** | -22.03(-31.03-1.49) | 0.19(-0.06-0.50) | 4.41(3.41-8.78)** | |  |
| 1920-1940 vs 1940-1960 | 0.74(0.54-0.96)** | 0.25(0.10-0.43)* | 0.00(-0.01-0.01) | 0.00(-0.01-0.01) | 0.99(1.79-2.21)** | |  |
| 1920-1940 vs 1960-1980 | 1.49(1.04-1.98)** | 0.51(0.23-0.83)** | -0.25(-0.50--0.04)* | -0.07(-0.11--0.04)** | 1.69(2.34-3.07)** | |  |
| 1920-1940 vs 1980-2000^a^ | 2.02(1.31-2.66)** | 24.5(0.81-33.77)** | -21.39(-27.52-1.94) | 0.22(0.10-0.33)** | 5.34(3.9-10.10)** | |  |
| 1940-1960 vs 1960-1980 | 0.45(0.16-0.74)* | 0.07(-0.10-0.26) | -0.10(-0.25-0.01) | -0.06(-0.10--0.03)** | 0.35(1.16-1.54)** | |  |
| 1940-1960 vs 1980-2000^a^ | 0.76(0.41-1.14)** | 11.32(0.22-15.78)* | -10.10(-13.32-0.81) | 0.19(0.08-0.31)** | 2.17(2.10-5.09)** | |  |
| 1960-1980 vs 1980-2000^a^ | 0.25(-0.15-0.64) | 29.39(0.07-39.87)* | -28.86(-38.22-0.46) | 0.59(0.19-1.16)* | 1.37(1.40-3.95)* | |  |
| Supplementary table 5. Results from four-way decomposition mediation analysis for low vs high, intermediate vs high and low vs intermediate education level across different birth year periods. *P value<0.05, **p value<0.001.  ^a^ Should be interpreted with caution due to the very low number of low-educated participants in 1980-2000, which leads to unpredictable Reference Interaction Effect (RIE) and Mediated Interaction Effect (MIE) values. | | | | | | | |
